# Materialistic Tendencies Lead to Less Empathy from Others

**DOI:** 10.3390/bs15050577

**Published:** 2025-04-25

**Authors:** Weinan Zeng, Yan Wang, Lijuan Cui, Ningning Feng

**Affiliations:** 1 School of Psychology and Cognitive Science, East China Normal University, Shanghai 200062, China; zengweinan@gdmu.edu.cn (W.Z.); wangyan@psy.ecnu.edu.cn (Y.W.); ljcui@psy.ecnu.edu.cn (L.C.); 2 Department of Psychology, School of Humanities and Management, Guangdong Medical University, Dongguan 523808, China

**Keywords:** empathy, materialism, perceived morality, perceived warmth

## Abstract

Empathy is crucial for social cohesion and prosocial behavior, yet the influence of a target’s materialism on observers’ empathy remains underexplored. This research investigates whether and how a target’s materialistic tendencies influence observers’ empathy, and the mechanisms underlying this effect. We proposed three hypotheses: (H1) observers exhibit less empathy for materialistic individuals compared to nonmaterialistic ones; (H2) perceived low morality mediates the negative effect of target materialism on empathy; and (H3) perceived lack of warmth also serves as a mediator. Across four studies, we tested these hypotheses. Study 1 (*n* = 190) found a significant difference in observers’ empathy toward high versus low materialistic targets. Study 2 (*n* = 362) demonstrated that this effect resulted from decreased empathy toward materialistic individuals rather than increased empathy toward nonmaterialistic ones, and together with Study 1, supported H1. Study 3 (*n* = 375) ruled out perceived social class as an alternative explanation, providing additional evidence for the independent effect of target materialism on empathy. Study 4 (*n* = 785) tested H2 and H3, and confirmed that perceived morality and perceived warmth both significantly mediated the effect of target materialism on observers’ empathy. These findings enhance our understanding of the negative social consequences of materialism and contribute to the literature on selective empathy and person perception.

## 1. Introduction

Imagine a scenario where a group of medical staff, during a year-end gathering, hangs a banner reading “The operating room is full of money”. Would you still be able to empathize with their joyful mood at the gathering? Or consider a young person who, in order to buy an iPhone, chooses to sell a kidney to raise funds—how would you evaluate this behavior? Another example is a female college student who frequently flaunts her luxury purchases on social media and, tragically, dies in a plane crash. Instead of sympathy, online users mock her. These events, though seemingly unrelated, are all based on real incidents that occurred in China in recent years. The common thread among them is the manifestation of materialism or excessive material pursuit, which also triggered negative reactions from the public. This raises an important question: if a person is perceived as materialistic, does it reduce others’ empathy towards them? To the best of our knowledge, this question has yet to be explored in the existing research.

Materialism is defined as a value orientation in which individuals place central importance on acquiring and possessing material goods, and regard them as essential to life satisfaction, personal success, and happiness (Richins & Dawson, 1992). According to this conceptualization, materialistic individuals not only prioritize material possessions in daily life, but also evaluate their sense of achievement and well-being through material ownership. Prior research has shown that such value orientations are associated with lower psychological well-being and impaired social functioning (Kasser & Ryan, 1993). Materialistic individuals tend to have lower life satisfaction and happiness (Jaspers et al., 2023; Ryan & Dziurawiec, 2001) and materialism has even been linked to an increased risk of depression (Azibo, 2013). Moreover, materialistic individuals are often viewed negatively by others, displaying more selfish and self-centered behaviors, which in turn reduces others’ willingness to interact with them (Van Boven et al., 2010). Materialists also exhibit fewer prosocial behaviors and are more likely to engage in aggressive actions (Lv et al., 2023). In terms of friendships, materialistic individuals are more likely to view relationships as a means to achieve material goals, often choosing friends who can elevate their social status or material gain, rather than those based on shared interests or emotional support (Purutçuoğlu & Doğan, 2017).

Regarding interpersonal dynamics, while progress has been made in understanding the consequences of materialism, there remain critical gaps in research. First, much of the existing literature focuses on how materialism harms the individuals who hold materialistic values (Kasser, 2016). However, less attention has been given to how materialistic individuals are perceived by others in social interactions. Second, despite findings showing that materialistic individuals tend to lack empathy towards others (Lv et al., 2023; Sheldon & Kasser, 1995), previous research has predominantly examined how individuals’ own materialistic values influence their empathy toward others—thus treating empathy primarily from the materialistic individual’s own perspective (i.e., empathy as an attribute of the materialistic person). In contrast, our study adopts an interpersonal perspective by treating materialistic individuals as the targets of empathy. Specifically, we investigate how observers’ empathy toward others changes when they perceive these others to be materialistic. This shift from a subject-centered to an object-centered perspective highlights an important, yet previously unexplored, dimension of interpersonal dynamics involving materialism. This study aims to address this gap by proposing that “the decline in empathy towards materialists occurs through two pathways: perceived morality and perceived warmth”.

We hypothesize that materialists are perceived as flawed in both moral dimensions (e.g., fairness, integrity) and social dimensions (e.g., warmth, cooperativeness). These negative evaluations, in turn, reduce observers’ motivation to engage in empathy (Zaki, 2014). We test these hypotheses through four studies.

### 1.1. The Observer’s Empathy Toward Materialists Is Influenced by Their Materialistic Traits

Empathy, defined as the ability to understand and share the emotional states of others (Davis, 1983), is the cornerstone of prosocial behavior and social cohesion. According to the motivation-driven model of empathy (Zaki, 2014), people’s empathy towards others is not automatically generated but is selectively regulated based on the characteristics of the target, the situation, and the individual’s motivations. In other words, people do not always express empathy towards others; they may reduce or avoid empathizing with others. The question now is: what factors may lead people to reduce their empathy towards others? Previous research has found that, for example, people typically empathize less with out-group members than with members of their own group (Cikara et al., 2011). Regarding the moral characteristics of the target individual, people tend to reduce empathy for those perceived as morally inferior (Riva et al., 2016; Wang et al., 2023). Some studies have even found that when misfortune befalls a morally lacking individual, people may experience more schadenfreude (Brambilla & Riva, 2017). Additionally, individuals in occupations that are frequently exposed to situations of suffering (e.g., healthcare workers) may also reduce their empathy towards others due to empathy fatigue (Sorenson et al., 2016).

These examples indicate that a variety of factors can lead to a reduction in empathy towards others. So, when someone is perceived as a materialist, will empathy towards them be influenced? As previously mentioned, materialists tend to be evaluated negatively by others (Van Boven et al., 2010) and exhibit a lack of prosocial behaviors in interpersonal interactions (Lv et al., 2023). More importantly, and directly relevant to this study, materialists themselves often show a lack of empathy towards others (Sheldon & Kasser, 1995). When facing others’ difficulties, empathizing is not always automatic because empathy involves emotional investment and cognitive effort (Scheffer et al., 2022). Individuals are likely to decide whether to empathize based on the perceived costs and benefits. Moreover, according to social exchange theory (Cropanzano & Mitchell, 2005), people engage in emotional and helping behaviors with others based on a reciprocal cost–benefit analysis. Due to the psychological and behavioral traits of materialists, others may have lower expectations of receiving high rewards in return. Therefore, when dealing with materialists, people are more likely to reduce their emotional investment and empathy. Based on this reasoning, we propose the first hypothesis of this study.

**H1:**  *Observers exhibit less empathy for materialistic individuals compared to nonmaterialistic ones.*

### 1.2. The Materialism of the Empathy Target Reduces Observer Empathy Through Perceived Morality and Perceived Warmth

The “Big Two Model” in social cognition provides a critical framework for understanding interpersonal evaluation mechanisms (Abele & Wojciszke, 2007). Subsequent research (Abele & Hauke, 2020; Abele et al., 2016) has further proposed that evaluations of others predominantly rely on two core dimensions of communion: morality (e.g., adherence to ethical norms such as trustworthiness and fairness) and warmth (e.g., capacity for emotional bonding, including traits like kindness and empathy). Grounded in this theoretical framework, the current study investigates observers’ evaluations of materialists and their empathic responses through the lens of these two dimensions.

Both morality and warmth play pivotal roles in shaping interpersonal impressions and relational dynamics. For instance, Brambilla et al. (2021) demonstrated that moral judgments dominate the entire process of impression formation, while Eisenbruch and Krasnow (2022) argued that warmth more effectively predicts long-term relational benefits. Given the centrality of morality and warmth in interpersonal evaluations, this study applies these dimensions to assess perceptions of materialists.

#### 1.2.1. Observers’ Perceived Morality of Materialists

Materialists may define their identity and success through material possessions, which leads them to be perceived as more self-centered and focused on personal gain rather than collective well-being (Kasser & Ryan, 1993). The perceived morality of materialists is nuanced and multifaceted. On the one hand, material success is seen as a positive and desirable goal in many cultures. On the other hand, excessive material pursuit may be viewed as greedy, selfish, or even a symbol of moral degradation (Muncy & Eastman, 1998; Yue et al., 2024).

The consumption choices and displays of wealth by materialists may be interpreted as neglecting social responsibility and moral obligations, showing a lack of concern for social fairness and environmental sustainability (Kasser, 2003). Numerous studies have explored the moral behavior and moral values of materialists, revealing that they tend to exhibit more antisocial behaviors (Cohen & Cohen, 2013), engage less in prosocial behavior (Briggs et al., 2007), have stronger social dominance orientations, hold more prejudiced beliefs about out-group members (Roets et al., 2006), are more likely to engage in questionable but legal behaviors (Lu & Lu, 2010), and are positively correlated with immoral consumption behaviors and attitudes (Gentina et al., 2018a, 2018b). Van Boven et al. (2010) examined the differences in social impressions and stereotypes between materialists and experiential seekers, finding that materialists generally receive more negative social evaluations and stronger social stereotypes than experiential seekers. Although much of the existing literature has focused on the moral and behavioral characteristics of materialists from their own perspective, research on the moral evaluation of materialists from the observer’s point of view remains limited. However, based on research findings on materialists’ behavior, motives, and social impressions, it can be hypothesized that the perceived morality of materialists may be lowered by observers.

Previous studies have shown that the moral level of the empathy target is a key factor in influencing empathy. When people perceive the target as having low moral standards, they tend to reduce their empathy for that person (Riva et al., 2016; Wang et al., 2023). Furthermore, some studies have found that when misfortune befalls a person with low morals, people may feel more schadenfreude, as they perceive it as a deserved outcome (Brambilla & Riva, 2017). This phenomenon of reduced empathy for morally inferior individuals is supported by neurophysiological and evolutionary psychology perspectives. For example, an ERP study found that compared to moral or neutral targets, when the target was immoral, the amplitude difference of the N2 component in response to painful and non-painful images became insignificant. This effect was attributed to a reduction in emotional arousal when observing the pain of an immoral person (Cui et al., 2016). Cui et al. (2016) suggest that, from an evolutionary perspective, our neural response to the pain of immoral people is diminished when facing potential danger, which increases our chances of survival. Based on this reasoning, we propose the second hypothesis of this study:

**H2:**  *Observers perceive low morality in individuals who exhibit materialistic tendencies, which in turn diminishes their empathy towards those individuals.*

#### 1.2.2. Observers’ Perceived Warmth of Materialists

Perceived warmth refers to the extent to which an individual is seen as friendly, kind, cooperative, and willing to establish connections with others. Research has shown that individuals with materialistic tendencies are more likely to exhibit self-centered and egoistic behaviors (Kasser, 2016), leading them to prioritize personal interests over pursuing collective benefits or developing deep interpersonal connections in social settings. This self-centered approach to social interactions may lower their evaluations in terms of cooperation, friendliness, and social skills. Furthermore, materialism is also associated with the scope and quality of one’s social network. Richins (1994) found that materialists typically have fewer social contacts, and these relationships tend to be of lower quality. This may be because materialists tend to invest more time and resources in the pursuit of material wealth, neglecting the development and maintenance of social relationships. Therefore, due to the intense material pursuit and resulting self-centered and egoistic behavior, materialists are likely to face negative feedback in perceived warmth. This negative feedback is primarily due to their shortcomings in cooperation, friendliness, social skills, and the quality and breadth of their social networks. As the empathy motivation-driven model proposed by Zaki (2014) suggests, people choose to express empathy based on personal motivations, goals, and values. From the perspective of social exchange theory, interpersonal relationships are viewed as exchange processes in which individuals seek to maximize their own benefits (Blau, 2017). In this framework, an individual’s perceived warmth could become an important factor in determining whether they are deemed worthy of empathy and emotional investment. Individuals with higher perceived warmth may be seen as more valuable social partners, and observers may be more inclined to show empathy towards them, fostering mutually beneficial relationships and long-term cooperation. In contrast, materialists, who receive lower perceived warmth ratings in terms of cooperation, friendliness, and other social attributes, may be seen as less deserving of emotional investment, thereby leading to reduced empathy toward them. Therefore, this study proposes the third hypothesis:

**H3:**  *Observers perceive less warmth in individuals who exhibit materialistic tendencies, which in turn diminishes their empathy towards those individuals.*

## 2. Research Overview

Across four studies, we examined the hypotheses regarding the effect of a target’s materialistic tendencies on observers’ empathy. In Study 1, we investigated the fundamental question of whether observers experience less empathy toward materialistic individuals compared to non-materialistic individuals. Study 2 introduced a control condition to disentangle whether target materialism reduces observers’ empathy or target non-materialism enhances it. In Study 3, we orthogonally manipulated both target materialism and social class to determine whether the effect of materialism on observers’ empathy operates independently of the target’s social class. Finally, in Study 4, we explored the mediating roles of perceived morality and warmth in this relationship. This study was approved by the University Committee on Human Research Protection (NO. HR1-0015-2023), and informed consent was obtained from all the participants prior to their participation.

## 3. Pretest 1: Development and Validation of Materials for Study 1

This pretest was conducted to evaluate the manipulation validity and clarity of the vignette materials to be used in Study 1. Each vignette consisted of two parts: the first described the target individual’s level of materialism (high vs. low), and the second presented a misfortune scenario involving income loss. One version of the vignette featured a high-materialism description paired with the misfortune event, while the other featured a low-materialism description paired with the same misfortune event. The two forms of vignettes contained 215 and 219 Chinese characters, respectively, with lengths kept consistent to control for differences in reading load. The materialism descriptions were developed based on the three core dimensions of the Material Values Scale (MVS) proposed by Richins and Dawson (1992): centrality, success, and happiness. These three dimensions served as the conceptual inclusion criteria for how materialism was portrayed and operationalized in our vignettes. We also referred to phrasing used in previous studies (e.g., Zhang & Mick, 2019) to ensure consistency with existing literature. To maintain conceptual clarity, we deliberately excluded features that, while potentially correlated with materialism, do not constitute its core definition. For example, materialism was not equated with wealth or socioeconomic status—a person may be wealthy without being materialistic, or vice versa. We also avoided conflating materialism with general consumption behaviors driven by utility or necessity. Additionally, we excluded constructs such as anti-consumerism or minimalism, which reflect ideological rejections of materialism but involve distinct motivational orientations. Instead, our manipulations focused specifically on whether the target character placed strong importance on acquiring material possessions and viewed them as essential to life satisfaction and personal success—consistent with the theoretical foundation of the MVS. The misfortune scenario—income reduction—was selected in line with commonly used manipulations in related research (e.g., Lucas & Kteily, 2018).

After drafting the vignettes, two psychology PhD holders independently assessed the alignment of each sentence with the three MVS dimensions to ensure adequate construct coverage. Their evaluations confirmed that the vignettes reflected the intended characteristics of materialism. Subsequently, we recruited an independent sample of 97 participants via the “Credamo” survey platform (similar to Mturk). The participants read both vignettes in a randomized order and rated the degree of materialism expressed in each using an 11-point Likert scale (0 = not at all, 10 = extremely high).

A paired-sample *t*-test revealed a significant difference between the two conditions: *M*_high_ = 8.51, *SD* = 1.16; *M*_low_ = 2.10, *SD* = 1.89; Mean difference = 6.40, *t*(96) = 27.37, *p* < 0.001, Cohen’s *d* = 2.78. These results indicate that the participants clearly distinguished between the high and low materialism conditions, supporting the strong manipulation validity of the materials for use in subsequent experimental tasks.

## 4. Study 1

Study 1 aimed to preliminarily test how the materialistic characteristics of the empathic target affect the observer’s empathy response, and to test the first hypothesis of this study: that the materialism of the empathic target will affect the observer’s empathy.

### 4.1. Method

#### 4.1.1. Research Design

Study 1 employed a single-factor, two-level between-subjects design, comparing participants’ empathy levels toward high-materialistic versus low-materialistic targets. The participants were randomly assigned to one of two experimental conditions: the high-materialistic target group or the low-materialistic target group. An independent samples *t*-test was used to compare mean differences in empathy scores between these two conditions.

The required sample size was estimated using G*Power 3.1 software, with a medium expected effect size (Cohen’s *d* = 0.5), a significance level (α) of 0.05, and statistical power (1 − *β*) of 0.80. The calculation indicated that each group required 64 participants, resulting in a total minimum sample size of *N* = 128. Considering potential data loss and unforeseen factors, we planned to recruit approximately 200 participants.

#### 4.1.2. Participants

A total of 200 questionnaires were distributed on the “Credamo” survey platform. Ten participants failed to answer one or two attention check questions correctly, and therefore their responses were excluded from the analysis as invalid. The remaining 190 responses included 75 males (39.5%) and 115 females (60.5%). All the participants were over 18 years old. Among them, 5.3% were aged 18–20, 37.4% were aged 21–30, 41.6% were aged 31–40, 7.4% were aged 41–50, 6.8% were aged 51–60, and 1.6% were aged 60 and above. The participants were randomly assigned to one of the two empathy target conditions: high-materialistic (*n* = 98) and low-materialistic (*n* = 92).

#### 4.1.3. Procedure and Materials

The participants read a character story, with each group receiving a different version of the story to manipulate the independent variable. A manipulation check followed to ensure the effectiveness of the independent variable manipulation. To check the manipulation of materialism, participants were asked, “Based on your understanding of materialism, how materialistic do you think Zhang Hua is?” (the participants rated on a scale from 0 to 10, with 10 representing the highest level of materialism). Additionally, there were two attention check questions: “*What difficulties did Zhang Hua face according to the materials above? (Multiple choice: 1. Divorce 2. Reduced income 3. Consumption ability affected)” and “This question checks whether you are answering seriously, please select 1*”. Finally, the participants answered five empathy measurement questions based on the character story and their personal feelings, as well as questions about their gender and age. After completing the experiment, the participants clicked “Submit” and received compensation via the platform. The entire experiment took about 5 min.

Both stories presented to the participants featured a character named Zhang Hua (a commonly used gender-neutral Chinese name, selected to minimize potential gender-related bias). In the high-materialism condition, the participants read about Zhang Hua exhibiting strong materialistic tendencies, whereas in the low-materialism condition, Zhang Hua was depicted as less materialistic. Following the character introduction, both versions described how Zhang Hua was recently affected by a salary cut, which impacted their daily spending (see Appendix A).

#### 4.1.4. Measures

The empathy measure was adapted from (Lucas & Kteily, 2018) study. Five questions were designed to assess the participants’ empathy toward the character, using a 7-point Likert scale. The internal consistency reliability (Cronbach’s α) for these five items was 0.932. For the materials in the two experimental groups, the internal consistency reliability (Cronbach’s α) was 0.951 and 0.815, respectively.

### 4.2. Results

#### 4.2.1. Manipulation Check and Attention Check

The manipulation check for materialism revealed a significant main effect, *t*(186.46) = 40.82, *p* < 0.001. The average materialism rating for the character in the high-materialism group was 8.76 (*SD* = 1.33, 95% CI [8.48, 9.01], *N* = 98), significantly higher than the average rating of 1.42 (*SD* = 1.14, 95% CI [1.20, 1.66], *N* = 92) in the low-materialism group. This indicates successful manipulation of the independent variable, as the participants’ ratings of the character’s materialism were consistent with the study’s design. Regarding attention checks, 10 participants failed to correctly answer one or more questions, and their responses were excluded from the analysis as invalid.

#### 4.2.2. Independent Samples *t*-Test Results

To assess the effect of the empathic target’s materialism on the observer’s empathy response, an independent samples *t*-test was conducted comparing the high- and low-materialism groups. The results indicated that observer empathy scores were significantly lower in the high-materialism group (*M* = 24.65, *SD* = 8.05, *N* = 98) than in the low-materialism group (*M* = 28.96, *SD* = 4.10, *N* = 92), *t*(146.17) = 4.69, *p* < 0.001. The effect size was substantial, with Cohen’s *d* = 0.67, suggesting that the materialistic traits of the target had a meaningful impact on the observer’s empathy.

## 5. Study 2

In Study 1, we preliminarily examined how different levels of materialism in empathy targets (high vs. low materialism) affected observers’ empathy. However, the finding that the observers exhibited varying degrees of empathy due to the materialism levels of the characters cannot definitively prove whether this difference is because observers increased their empathy for low materialists, decreased their empathy for high materialists, or whether both effects occurred simultaneously. Therefore, in Study 2, we introduce a reference group as a baseline. By comparing the empathy responses of the high- and low-materialism groups to those of the reference group, we aim to determine whether the observed differences in empathy are due to an increase in empathy for low materialists, a decrease in empathy for high materialists, or both.

### 5.1. Method

#### 5.1.1. Research Design

Study 2 employed a single-factor, four-level between-subjects design. The participants were randomly assigned to one of four groups based on the materialism characteristics presented in the vignette: high-materialism, moderate-materialism, low-materialism, and no-materialism-description. A one-way analysis of variance (ANOVA) was used to examine mean differences in observers’ empathy across the four conditions.

The required sample size was estimated using G*Power, with an expected medium effect size (Cohen’s *f* = 0.25), a significance level (*α*) of 0.05, and a desired statistical power of 0.80 for four groups. The calculation indicated a minimum sample size of 136 participants (34 per group). To account for possible data loss and to ensure sufficient statistical power for post hoc comparisons, we recruited a total of 400 participants.

#### 5.1.2. Participants

A total of 400 questionnaires were distributed through the “Credamo” survey platform. In terms of attention checks, 38 participants failed to answer one or more attention-check questions correctly, and their responses were excluded from the statistical analysis. The remaining 362 valid responses were included in the analysis. Among the participants, 109 were male (30.1%) and 253 were female (69.9%). All the participants were 18 years or older, with the following age distribution: 18–20 years (6.1%), 21–30 years (46.1%), 31–40 years (37.0%), 41–50 years (7.5%), 51–60 years (2.8%), and over 60 years (0.6%). Based on the materialism characteristics assigned to the character in the story, the participants were randomly divided into four groups: the high-materialism group (*N* = 94), the moderate-materialism group (*N* = 93), the low-materialism group (*N* = 88), and the no-materialism-description group (*N* = 87).

#### 5.1.3. Procedure and Materials

The participants read a character story, with the four groups reading four different versions of the story, serving as the manipulation of the independent variable. Following this, the manipulation check for materialism was performed by asking, “*Based on your understanding of materialism, how materialistic do you think the character Zhang Hua is*?” (the participants rated on a scale from 0 to 10, where 10 represents the highest level of materialism). Attention checks were also included, as follows: “*What difficulties is Zhang Hua currently facing, as mentioned in the material*?” (*Multiple choice: 1. Divorce 2. Reduced income 3. Consumption ability affected*) and “*Please select 2 for this attention check*” (note: for the “no-materialism” reference group, no materialism-related characteristics were mentioned in the character story, so the materialism manipulation check was not included). Then, the participants answered five empathy measurement questions based on the presented character story and their personal feelings, followed by questions on their gender and age. Finally, the participants clicked “Submit” to complete the experiment, and the platform provided compensation. The entire experiment took approximately 5 min.

The materials for the high- and low-materialism groups were the same as in Study 1. The materials for the moderate-materialism group reflected the character’s moderate materialism, while the “no-materialism” group received a version where materialism-related characteristics were not mentioned (see Appendix A). To accommodate the moderate-materialism condition introduced in Study 2, we developed a new vignette designed to represent an intermediate level of materialism. The construction of this vignette followed the same theoretical framework as in Pretest 1, based on the three core dimensions of the Material Values Scale (Richins & Dawson, 1992): centrality, success, and happiness. Unlike the high- or low-materialism vignettes, the moderate-materialism version described the character as valuing material possessions in a balanced way—acknowledging their utility and partial relevance to life satisfaction, without portraying them as central to identity or success. To ensure that this vignette effectively conveyed a moderate level of materialism, we conducted a pretest using the same procedure as in Pretest 1. A separate sample of 96 participants was recruited via the Credamo platform and asked to rate the perceived materialism of the vignette using an 11-point Likert scale (0 = not at all, 10 = extremely high). The results showed a mean rating of *M* = 4.80, *SD* = 1.45, on an 11-point Likert scale ranging from 0 to 10. This score is close to the midpoint (5), indicating that the vignette was perceived as representing a moderate level of materialism, effectively falling between the high- and low-materialism conditions and confirming the validity of the manipulation. Therefore, this vignette was deemed valid for inclusion in Study 2.

#### 5.1.4. Measures

The dependent variable, observer empathy, was measured using the same method as in Study 1. In Study 2, the internal consistency reliability (Cronbach’s α) for the five empathy questions was 0.811. The internal consistency for the four experimental groups (high, moderate, low, and none) was 0.853, 0.783, 0.814, and 0.599, respectively.

### 5.2. Results

#### 5.2.1. Manipulation Check and Attention Check

The main effect for the materialism manipulation check was significant, *F*(2, 272) = 362.14, *p* < 0.001. The average ratings of materialism for the high-, moderate-, and low-materialism groups were 8.30 (*SD* = 1.335, 95% CI [8.02, 8.57], *N* = 94), 4.73 (*SD* = 1.825, 95% CI [4.36, 5.11], *N* = 93), and 2.07 (*SD* = 1.507, 95% CI [1.75, 2.39], *N* = 88), respectively. These results confirm that the manipulation of the independent variable was effective, as the participants’ ratings of the materialism of the character were consistent with the study’s design. (Note: For the “no-materialism” reference group, as no materialism characteristics were described, the materialism manipulation check was not applied.) In the attention checks, 38 participants failed to answer one or more attention check questions correctly, and their responses were excluded from the statistical analysis.

#### 5.2.2. ANOVA Results

As shown in Table 1, a one-way ANOVA with observer empathy as the dependent variable and materialism level (low/medium/high/none) as the independent variable revealed a significant main effect of materialism on empathy, *F*(3, 358) = 16.46, *p* < 0.001, η_p_^2^ = 0.121. Descriptive statistics showed that the high-materialism group had the lowest empathy score (*M* = 23.97, *SD* = 5.54), while the “no-materialism” group had the highest empathy score (*M* = 28.38, *SD* = 3.01). The moderate-materialism group had a mean score of 26.49 (*SD* = 4.52), and the low-materialism group had a mean score of 27.08 (*SD* = 3.84). Post-hoc comparisons showed that the high-materialism group scored significantly lower on empathy than the low-materialism group, with a mean difference of 3.11 (95% CI [1.84, 4.38]), *p* < 0.001, consistent with the findings of Study 1. Compared to the moderate-materialism and no-materialism groups, the high-materialism group also showed significantly lower empathy scores, with mean differences of 2.52 (95% CI [1.26, 3.77]), *p* < 0.001, and 4.41 (95% CI [3.14, 5.69]), *p* < 0.001, respectively. The difference in empathy scores between the low-materialism group and the moderate-materialism group was not significant, although the difference with the no-materialism group approached significance (*p* = 0.049, mean difference = 1.30, 95% CI [0.00, 2.60]). However, since the material length of the no materialism group was significantly different from the other groups, and the internal consistency reliability of the empathy scale (Cronbach’s α = 0.599) in this group was notably lower than in the other groups, we primarily use the moderate-materialism group as the reference. Therefore, it can be concluded that the observer reduced their empathy toward high materialists, rather than increasing their empathy toward low materialists.

### 5.3. Discussion (Studies 1 and 2)

Study 1 and Study 2 were designed to test Hypothesis 1 (H1), which posits that observers exhibit less empathy for materialistic individuals compared to nonmaterialistic ones. Study 1 provided initial empirical evidence, which was subsequently refined and clarified by the design and results of Study 2, thus we discuss these two studies together to clearly convey their logical interconnectedness and incremental contributions. Both studies support H1 by demonstrating that the materialism of the target of empathy influences the observer’s empathic response. Specifically, the results of Study 1 revealed a significant effect of the target’s materialism level on observers’ empathy. Compared to low-materialism targets, high-materialism targets received significantly lower empathy. This result suggests that, like other characteristics of the target (such as gender, age, and race) (Deska et al., 2020; Horgas & Elliott, 2004; Riva et al., 2016), the materialistic values of the target also affect the observer’s empathic response. This finding offers preliminary support for H1, laying the empirical groundwork for deeper examination in subsequent studies.

Study 2 extended the test of H1 by including two control groups to determine whether the observed empathy gap between high and low materialists was driven by increased empathy toward low materialists, decreased empathy toward high materialists, or both. The results revealed that high materialists received significantly lower empathy scores than all the other groups, while the empathy scores for low materialists did not significantly differ from either of the two control groups. This result indicates that the observed empathy difference toward high and low materialists was primarily due to a reduction in empathy for high materialists, rather than an increase in empathy for low materialists. This finding reflects that the difference in the observer’s empathy toward high and low materialists comes mainly from the decreased empathy for high materialists. This result is consistent with the study by Van Boven et al. (2010), where they also found that people’s negative impressions of materialistic behaviors were more due to devaluing materialism rather than appreciating experientialism. Similarly, the study by Riva et al. (2016) found that the differences in empathy for individuals with varying moral levels were due to a reduction in empathy for low-morality targets rather than an increase in empathy for high-morality targets.

## 6. Study 3

In Studies 1 and 2, we examined the effect of the materialism of the empathy target on the observer’s empathy, demonstrating that the level of materialism in the empathy target influences the observer’s empathic response. However, alternative explanations for this finding may exist. Since the concept of materialism is largely associated with money and wealth, it naturally connects to income and social class (although materialism itself represents a value system and is not the same as these factors). Previous research has shown that people’s empathy responses differ across social classes (Lucas & Kteily, 2018; Matos Devesa, 2022). Therefore, an alternative explanation could be that the differences in observers’ empathy toward empathy targets with varying levels of materialism might result from perceived differences in the target’s social class. The question then arises: if social class is controlled as a variable, does the influence of materialism on empathy remain significant?

Study 3 aimed to analyze the independent and interactive effects of the empathy target’s materialism and social class on the observer’s empathy response. Specifically, we sought to verify whether, after controlling for differences in social class, the materialism of the empathy target would still independently influence the observer’s empathy. This investigation is based on previous research findings indicating that both materialism and social class are crucial factors influencing interpersonal evaluations and emotional responses. However, how these two factors independently and jointly influence the empathy process remains an open question. In Study 3, we manipulated both materialism and social class to examine whether the materialism of the empathy target continues to exert an independent effect on the observer’s empathy after accounting for the influence of social class.

### 6.1. Methods

#### 6.1.1. Research Design

Study 3 employed a 2 (target materialism: high vs. low) × 2 (target social class: high vs. low) between-subjects factorial design. A two-way analysis of variance (ANOVA) was used to examine both main effects and interaction effects between materialism and social class on observers’ empathy responses.

The sample size was determined using G*Power software, with parameters set as follows: effect size f = 0.25 (medium), significance level α = 0.05, statistical power = 0.80, and four experimental conditions (*df* for interaction = 1). The estimated minimum sample size was 128 participants (32 per group). Given this study’s focus on detecting interaction effects and to enhance statistical robustness, we recruited 400 participants.

#### 6.1.2. Participants

A total of 400 questionnaires were distributed via the “Credamo” survey platform. In the attention check, 25 participants failed to correctly answer one or more of the attention-check questions, and their responses were excluded from the analysis. The remaining 375 valid responses were included in the final dataset. Among the participants, 137 were male (36.5%) and 238 were female (63.5%). All the participants were aged 18 and above, with the following age distribution: 18–20 years (6.7%), 21–30 years (44.3%), 31–40 years (36.3%), 41–50 years (6.4%), 51–60 years (5.3%), and over 60 years (1.1%). Each participant was randomly assigned to one of the four experimental groups, with each group consisting of 94, 96, 88, and 97 participants, respectively.

#### 6.1.3. Procedure and Materials

The participants then read a character story, with each group reading a different version of the story to manipulate the independent variables. Following this, a manipulation check was conducted. The manipulation check for social class asked the participants, “*Based on Zhang Hua’s job and income, where do you think Zhang Hua falls in the social class hierarchy*?” (rated from 0 to 10, with 10 representing the highest social class). The manipulation check for materialism asked, “*Based on your understanding of materialism, how materialistic do you think Zhang Hua is*?” (rated from 0 to 10, with 10 representing the highest level of materialism).

Additionally, two attention check questions were included: “*What difficulties is Zhang Hua currently facing, as mentioned in the material*?” (multiple-choice: 1. divorce, 2. reduced income, and 3. decreased purchasing power). This question was to check if the participants were paying attention. The participants were asked to please select 1. Afterward, the participants answered five empathy measurement questions based on the presented character story and their personal feelings, as well as demographic questions about their gender and age. Finally, the participants clicked “Submit” to complete the experiment, and the platform distributed compensation. The entire experiment took approximately 5 min.

Study 3 employed a 2 (materialism of the empathy target: high vs. low) × 2 (social class of the empathy target: high vs. low) between-subjects factorial design. The participants were randomly assigned to one of four groups: high-social-class–high-materialism (*n* = 94), high-social-class–low-materialism (*n* = 96), low-social-class–high-materialism (*n* = 88), or low-social-class–low-materialism (*n* = 97), with each group reading one version of the character story (see Appendix A).

#### 6.1.4. Measures

The dependent variable was observer empathy, measured using the same method as in the previous studies. In Study 3, the internal consistency reliability (Cronbach’s α) of the observer empathy scale was 0.882, with the internal consistency reliability for the four experimental conditions being 0.873, 0.887, 0.894, and 0.799, respectively.

### 6.2. Results

#### 6.2.1. Manipulation Check and Attention Check

The manipulation check for social class showed a significant main effect, *F*(1, 373) = 558.64, *p* < 0.001. The participants in the high-social-class conditions rated the character’s social class significantly higher (*M* = 8.04, *SD* = 1.36, 95% CI [7.85, 8.24], *N* = 190) than those in the low-social-class conditions (*M* = 3.75, *SD* = 2.09, 95% CI [3.45, 4.05], *N* = 185).

The manipulation check for materialism also showed a significant main effect, F(1, 373) = 571.73, *p* < 0.001. The participants in the high-materialism conditions rated the character’s materialism significantly higher (*M* = 7.86, *SD* = 2.04, 95% CI [7.56, 8.16], *N* = 182) than those in the low-materialism conditions (*M* = 2.59, *SD* = 2.21, 95% CI [2.28, 2.90], *N*= 193).

These results indicate that the manipulation of the independent variables was effective, as the participants’ evaluations of the character’s social class and materialism aligned with the intended study design.

Regarding the attention check, 25 participants failed to correctly answer one or more of the attention-check questions. Their responses were excluded from the statistical analysis as invalid.

#### 6.2.2. ANOVA Results

A two-way ANOVA was conducted with observer empathy as the dependent variable and materialism level and social class of the empathy target as the independent variables. This analysis examined the main effects of the two independent variables on the dependent variable, as well as their interaction effect. If only the main effects were significant, but the interaction effect was not, it would indicate that the two independent variables influenced the dependent variable independently.

As shown in Table 2, the results revealed a significant main effect of materialism on observer empathy, *F*(1, 371) = 19.00, *p* < 0.001, η_p_^2^= 0.049. Specifically, the low-materialism group had a higher mean empathy score (*M* = 25.95, *SD* = 5.94) compared to the high-materialism group (*M* = 23.12, *SD* = 6.79). This finding indicates that observers exhibited significantly lower empathy toward high-materialism targets than low-materialism targets, consistent with the results of the previous two studies, thus supporting Hypothesis 1.

Similarly, the main effect of social class on observer empathy was also significant, *F*(1, 371) = 18.63, *p* < 0.001, η_p_^2^ = 0.048. The low-social-class group had a higher mean empathy score (*M* = 26.02, *SD* = 6.10) than the high-social-class group (*M* = 23.17, *SD* = 6.62). This result suggests that there was a significant difference in empathy based on the target’s social class, with observers showing significantly lower empathy toward high-social-class targets compared to low-social-class targets.

However, the interaction effect between materialism and social class was not statistically significant, *F*(1, 371) = 2.29, *p* = 0.132, η_p_^2^ = 0.006. This indicates that the two independent variables did not significantly interact with each other.

Therefore, these findings suggest that the effect of the empathy target’s materialism on observer empathy is independent of social class, ruling out social class as an alternative explanation for the results.

### 6.3. Discussion

Study 3 was designed to further test Hypothesis 1 (H1)—that observers exhibit lower empathy toward individuals perceived as materialistic—by examining whether this effect could be explained by a confounding factor: the perceived social class of the empathy target. Although materialism and social class are two distinct concepts, materialism pertains to attitudes toward wealth and money, while social class inherently includes wealth and financial resources. This overlap may lead to misunderstandings regarding the relationship between the two constructs.

To rule out social class as an alternative explanation for our experimental results, we included “the social class of the empathy target” as an additional independent variable alongside the original materialism variable. The results of the two-way ANOVA indicated that both materialism and social class of the empathy target had significant main effects on observer empathy. Specifically, observer empathy was influenced by the social class of the target, with lower empathy being shown toward high-social-class targets compared to low-social-class targets. This finding is consistent with previous research (Guo et al., 2012; Lucas & Kteily, 2018).

However, there was no significant interaction effect between materialism and social class, suggesting that even after controlling for the empathy target’s social class, materialism still independently influenced observer empathy. In other words, the observed differences in empathy toward targets with different levels of materialism cannot be explained by the target’s social class. Therefore, we can confidently rule out social class as a potential alternative explanation for our findings.

These findings provide additional support for H1, reinforcing the conclusion drawn from Studies 1 and 2 that perceived materialism alone is sufficient to reduce empathic responses, regardless of the target’s socioeconomic background. This strengthens the validity of our manipulation and rules out social class as a plausible alternative explanation for the empathy gap observed in earlier studies. However, the underlying psychological mechanisms behind why observers reduce their empathy toward high-materialism targets remain unclear. Study 4 will further explore these mechanisms in greater depth.

## 7. Pretest 2: Development and Validation of New Materials for Study 4

To strengthen the robustness of the experimental manipulation and avoid potential construct contamination, we developed a new set of vignettes for Study 4. This included revised high- and low-materialism descriptions and a broader set of misfortune scenarios. Rather than reusing the same textual materials from earlier studies, we intentionally varied the wording while retaining the core theoretical structure of materialism. This approach aimed to reduce semantic repetition and ensure that the observed effects were not dependent on a single linguistic formulation or scenario. It also supports the conceptual purity of the construct manipulation.

In addition, we developed ten distinct misfortune scenarios covering diverse domains of adversity, such as emotional loss, financial hardship, health issues, and unexpected disruptions. Introducing multiple types of negatively valenced life events allowed us to better simulate real-world complexity, increase the ecological validity of the design, and improve the generalizability of our findings across different emotional contexts.

### 7.1. Development of Materialism Vignettes

The new high- and low-materialism descriptions were constructed in line with the three core dimensions of the Material Values Scale (Richins & Dawson, 1992): centrality, success, and happiness. The high-materialism vignette portrayed a person who places strong emphasis on material possessions as a source of identity and life satisfaction, frequently displays possessions to signal success, and prioritizes material consumption even at the expense of other life domains. Conversely, the low-materialism vignette described a person who values inner experience, avoids conspicuous consumption, and believes happiness comes from emotional, spiritual, and social fulfillment. These descriptions maintained the theoretical integrity of the materialism construct while introducing a new linguistic framing to strengthen the manipulation’s generalizability.

### 7.2. Development and Selection of Misfortune Scenarios

We initially created 10 vignette-style misfortune scenarios involving a single character, Zhang Hua, who experiences a negative event. These events included a stolen phone, failed investment, chronic illness, lost photo, pet death, family health crisis, relationship failure, canceled travel, car accident, and job insecurity. These scenarios were designed to vary in theme while maintaining a comparable level of emotional negativity, and to be easily understood by participants from different backgrounds.

To select the final set of scenarios for use in the main study, we applied two criteria: (1) Moderate to high levels of perceived emotional negativity, based on participant ratings; (2) Thematic diversity, to ensure coverage of various real-life domains (e.g., health, finance, sentimentality, and privacy). Scenarios that scored relatively low in perceived negativity or showed thematic redundancy were excluded. Based on these criteria, four scenarios were selected: Scenario 5: phone theft and privacy loss; Scenario 7: loss of a meaningful photo; Scenario 8: investment failure; and Scenario 10: chronic illness diagnosis.

### 7.3. Validation Procedure

To validate the newly developed materials, we recruited an independent sample of 97 participants via the Credemo platform. Each participant read the high- and low-materialism vignettes (randomized order) and rated the perceived materialism of the character on an 11-point Likert scale (0 = not at all materialistic, 10 = extremely materialistic). They also rated the negativity or positivity of each misfortune scenario on a similar scale (0 = extremely negative, 10 = extremely positive), where lower scores indicate higher perceived emotional negativity.

### 7.4. Results

A paired-sample *t*-test showed a statistically significant difference in perceived materialism between the high- and low-materialism descriptions: high-materialism vignette: *M* = 8.68, *SD* = 1.43; low-materialism vignette: *M* = 2.35, *SD* = 1.99; and mean difference = 6.33, *t*(96) = 25.92, *p* < 0.001, and Cohen’s *d* = 2.41. This result confirmed the strong and distinct manipulation of materialism perception.

The four selected scenarios (Scenarios 5, 7, 8, and 10) all received low mean scores, confirming their effectiveness in eliciting negative emotional responses. The descriptive statistics were as follows: *M*_5_ = 1.61, *SD*_5_ = 1.34; *M*_7_ = 2.40, *SD*_7_ = 1.78; *M*_8_ = 1.93, *SD*_8_ = 1.36; *M*_10_ = 1.88, and *SD*_10_ = 1.58. The thematic variation across these events ensured a broad coverage of emotional domains, supporting their suitability for use in the main experiment.

### 7.5. Conclusion

Together, the results confirmed the validity and clarity of the newly developed high- and low-materialism vignettes and the selected misfortune scenarios. These materials were thus considered suitable for use in Study 4, providing a linguistically distinct yet conceptually consistent manipulation of materialism and a realistic emotional context for empathy elicitation.

## 8. Study 4

Study 4 was conducted based on stereotype theory, aiming to examine the mediating roles of perceived morality and perceived warmth in the effect of the materialism of the empathy target on observer empathy. This study tested Hypotheses 2 and 3.

### 8.1. Method

#### 8.1.1. Research Design

Study 4 employed a single-factor, two-level between-subjects design, aiming to investigate the underlying mechanisms linking target materialism to observers’ empathy. The participants were randomly assigned to either a high-materialism or low-materialism condition.

We proposed two parallel mediating variables (perceived morality and perceived warmth) to explain how target materialism influenced empathy levels. To test this parallel mediation model, we employed the Bootstrap method recommended by Hayes (2017). Specifically, PROCESS Macro (Model 4) was used with 5000 bootstrap samples to estimate indirect effects and construct 95% confidence intervals for the mediation effects of perceived morality and perceived warmth. Mediation effects were considered significant if the confidence intervals excluded zero.

Given the statistical stability requirements of the Bootstrap procedure (Hayes, 2017) and recommendations to control collinearity and ensure adequate statistical power in parallel mediation models (Fritz & MacKinnon, 2007), the ideal sample size was determined to exceed 200 participants. To further ensure statistical robustness, we aimed to recruit around 800 participants.

#### 8.1.2. Participants

A total of 840 questionnaires were distributed via the “Credamo” survey platform. Among the respondents, 55 participants failed to answer the attention check question correctly, and their responses were excluded from the analysis. The final dataset included 785 valid responses, consisting of 257 males (32.7%) and 528 females (67.3%). All the participants were aged 18 and above, with the following age distribution: 18–20 years: 5.5%, 21–30 years: 41.4%, 31–40 years: 42.3%, 41–50 years: 6.6%, 51–60 years: 3.3%, 60 years and above: 0.9%.

#### 8.1.3. Procedure and Materials

The participants first read a description of the character’s materialistic traits, with each group receiving a different version (high vs. low materialism) as a manipulation of the independent variable. Afterward, a manipulation check was conducted by asking the participants the following question: “*Based on your understanding of materialism, how materialistic do you think Zhang Hua is*?” (rated on a 0–10 scale, with 10 representing the highest level of materialism). Next, the participants completed the moral evaluation and social evaluation scales, providing ratings for the character. Then, each participant was randomly assigned to read one of four vignettes describing a recent misfortune that Zhang Hua experienced. Following the vignette, an attention check question was presented: “*What difficulties is Zhang Hua currently facing, as mentioned in the material*?”. The participants then answered five empathy measurement questions based on their perception of the character, as well as demographic questions regarding gender and age. Finally, the participants clicked “Submit” to complete the experiment, and the platform distributed compensation. The entire experiment took approximately 7 min. Details of the character descriptions and vignettes can be found in Appendix A.

#### 8.1.4. Measures

Observer empathy was measured using the same method as in the previous studies. In Study 4, the internal consistency reliability (Cronbach’s α) for the five-item observer empathy scale was 0.902.

Perceived morality and perceived warmth were measured using the moral evaluation scale and social evaluation scale developed by Jieting et al. (2015).

For the moral evaluation scale, the participants rated the character based on four attributes: “*frank and straightforward*”, “*honest and sincere*”, “*trustworthy*”, and “*loyal and honest*”.

For the social evaluation scale, the participants rated the character based on six attributes: “*likeable*”, “*friendly and approachable*”, “*understanding and empathetic*”, “*willing to help others*”, “*enthusiastic and warm-hearted*”, and “*responsible*”.

Ratings were given on a 5-point Likert scale, ranging from “*strongly disagree*” to “*strongly agree*”, based on the observer’s perception. In this study, the internal consistency reliability (Cronbach’s α) for the moral evaluation scale was 0.903, and for the social evaluation scale, it was 0.946.

### 8.2. Results

#### 8.2.1. Manipulation Check and Attention Check

The manipulation check for materialism showed a significant main effect, *t*(783) = 71.15, *p* < 0.001, with a Cohen’s *d* of 5.08. The participants in the high-materialism condition rated the character’s materialism significantly higher (*M* = 8.60, *SD* = 1.40, 95% CI [8.45, 8.73], *N* = 396) compared to participants in the low-materialism condition (*M* = 1.42, *SD* = 1.42, 95% CI [1.29, 1.57], *N* = 389), with a mean difference of 7.18.These results indicate that the manipulation of the independent variable was successful, as the participants’ evaluations of the character’s materialism aligned with the intended study design. Regarding the attention check, 55 participants failed to correctly answer the attention check question, and their responses were excluded from the statistical analysis.

#### 8.2.2. ANOVA Results

In Study 4, we conducted a multivariate analysis of variance (MANOVA) to assess the effects of the materialism level of the empathy target (high vs. low) on the observer’s perception of the target’s morality, warmth, and empathy (see Table 3).

(1) Perceived Morality

The results showed that the materialism level of the empathy target had a significant effect on the observer’s perception of morality, *F*(1, 783) = 524.23, *p* < 0.001, and η*_p_*^2^ = 0.401. The participants in the high-materialism condition rated the character significantly lower on moral evaluation (*M* = 11.82, *SD* = 4.06) than those in the low-materialism condition (*M* = 17.06, *SD* = 1.99), indicating that observers tended to assign lower moral ratings to high-materialism individuals.

(2) Perceived Warmth

A similar pattern was observed in perceived warmth. The materialism level of the empathy target had a significant effect on observer’s perception of warmth, *F*(1, 783) = 577.98, *p* < 0.001, η*_p_*^2^ = 0.425. The participants in the high-materialism condition rated the character significantly lower in warmth evaluation (*M* = 17.23, *SD* = 6.09) than those in the low-materialism condition (*M* = 25.50, *SD* = 3.01), suggesting that observers perceived high-materialism individuals as less warmth.

(3) Observer Empathy

The effect of the materialism level of the empathy target on observer empathy was also significant, *F*(1, 783) = 119.60, *p* < 0.001, and η*_p_*^2^ = 0.133. The participants in the high-materialism condition exhibited significantly lower empathy toward the character (*M* = 25.42, *SD* = 6.49) compared to those in the low-materialism condition (*M* = 29.41, *SD* = 3.14). This result indicates that observers displayed lower levels of empathy toward high-materialism individuals.

Overall, these results consistently support our hypothesis, showing that the empathy target’s materialism level significantly influenced the observer’s perception of morality, warmth, and empathy. Specifically, compared to low-materialism individuals, high-materialism individuals were perceived as having lower morality and warmth, and observers exhibited lower empathy toward them.

#### 8.2.3. Mediation Analysis Results

To examine mediation effects, we conducted a mediation analysis using observer empathy as the dependent variable, materialism of the empathy target as the independent variable, and perceived morality and perceived warmth as mediating variables.

**Figure 1:**
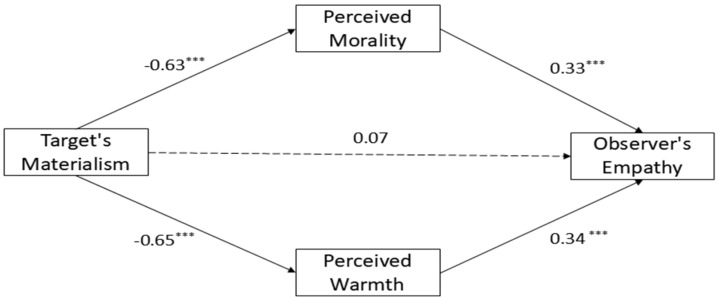
Perceived Morality and Perceived Warmth as Mediators in the Relationship between Materialism and Empathy (Study 4). ***: *p* < 0.001.

We used PROCESS macro (Model 4) developed by Hayes (2017), with 5000 bootstrap resamples. The results are presented in Figure 1.

- Mediation Path Analysis

A parallel mediation analysis using 5000 bootstrap resamples was conducted, with the following results (see Table 4):

(1) Mediation Effect of Perceived Morality

The materialism level of the empathy target significantly negatively predicted perceived morality (*β* = −0.63, *B* = −2.62, *SE* = 0.11, *t* = −22.90, and *p* < 0.001). In turn, perceived morality positively predicted observer empathy (*β* = 0.33, *B* = 0.43, *SE* = 0.09, *t* = 5.00, and *p* < 0.001). The indirect effect was −1.13 (BootSE = 0.25), with a 95% Bootstrap confidence interval of [−1.63, −0.65], which did not contain 0, indicating that the mediation effect of perceived morality was significant. The partially standardized indirect effect was *β* = −0.21 (BootSE = 0.04, 95% CI [−0.29, −0.12]), explaining 20.63% of the total effect (accounting for 48.11% of the total indirect effect).

(2) Mediation Effect of Perceived Warmth

The materialism level of the empathy target significantly negatively predicted perceived warmth (*β* = −0.65, *B* = −4.13, *SE* = 0.17, *t* = −24.04, and *p* < 0.001). In turn, perceived warmth positively predicted observer empathy (*β* = 0.34, *B* = 0.30, *SE* = 0.06, *t* = 5.14, and *p* < 0.001). The indirect effect was −1.22 (BootSE = 0.23), with a 95% Bootstrap confidence interval of [−1.70, −0.77], which did not contain 0, indicating that the mediation effect of perceived warmth was significant. The partially standardized indirect effect was *β* = −0.22 (BootSE = 0.04, 95% CI [−0.31, −0.14]), explaining 22.27% of the total effect (accounting for 51.89% of the total indirect effect).

(3) Total Indirect Effect

The total indirect effect was −2.35 (BootSE = 0.22, 95% CI [−2.79, −1.95]), accounting for 117.9% of the total effect (as the direct effect was reversed in direction).

This result confirms that the effects of materialism on empathy were fully mediated by perceived morality and perceived warmth.

### 8.3. Discussion

Study 4 was conducted to test Hypotheses 2 and 3, which propose that observers’ reduced empathy toward materialistic individuals is mediated by two mechanisms: H2, perceived low morality, and H3, perceived low warmth.

Our findings provide strong support for both hypotheses. First, the participants perceived high-materialism targets as significantly lower in morality compared to low-materialism targets. This result aligns with previous research findings on the moral characteristics of materialists. Muncy and Eastman (1998), in a study on consumer behavior, found that individuals with higher levels of materialism exhibited poorer consumer ethics. They were more likely to engage in unethical consumer behaviors such as fraud, theft, and resource misuse. When faced with unfair pricing or product quality issues, they tended to prioritize personal gain over ethical responsibility and the interests of others. Similarly, Chatterjee et al. (2000) found a negative correlation between materialism and altruism, suggesting that materialists are often more self-centered in social interactions and less concerned with the needs and well-being of others. Kasser (2003) also noted that materialists tend to exhibit lower moral standards, including dishonesty, selfishness, and unethical consumer behaviors. Additionally, Chowdhury and Fernando (2013) discovered that high-materialism consumers tend to score lower on moral beliefs and ethical consumer behaviors. The present study expands upon these findings by showing, from the observer’s perspective, that materialistic individuals elicit lower moral evaluations—providing empirical support for H2.

Second, high-materialism individuals were also perceived as lower in warmth compared to low-materialism individuals. This finding supports H3 and is consistent with previous research on the interpersonal characteristics of materialists. Solberg et al. (2004) argued that materialists tend to exhibit selfish, self-serving, and unempathetic behaviors in interpersonal relationships, which may damage the quality of their social interactions. Similarly, Van Boven et al. (2010) found that materialists are often perceived as selfish and unlikable, leading to negative evaluations and social discrimination in social interactions. Kasser (2003) also pointed out that materialism harms interpersonal relationships, causing individuals to focus excessively on personal gains while neglecting the needs and feelings of others.

Based on Zaki (2014) motivation-based model of empathy, individuals evaluate the potential psychological or material costs of expressing empathy—such as time, effort, emotional burden, and the potential benefits of social recognition, personal fulfillment, or strengthened social bonds—before deciding whether to empathize with others. This cost–benefit analysis influences whether individuals choose to engage in empathetic responses, ultimately determining the depth and scope of their empathy. Thus, in the case of materialists, their lower moral and social evaluations reduce observers’ willingness to extend empathy toward them. This finding highlights the role of materialism in shaping social exchange and empathy processes. Materialists, being perceived as lower in moral and social attributes, become less attractive social partners, ultimately influencing the level of empathy observers are willing to show them.

The mediation analysis further revealed that perceived morality and perceived warmth fully mediated the relationship between the materialism of the empathy target and the observer’s empathy, providing direct statistical support for H2 and H3. Therefore, we suggest that the reduction in empathy toward materialists is mediated by perceptions of lower morality and reduced social warmth.

## 9. General Discussion

Across four studies, the present research systematically investigated whether and how the materialistic orientation of an empathy target influences the empathy expressed by observers. The results consistently supported our central prediction: materialistic individuals elicit lower empathy from others. Study 1 provided initial evidence for H1 that high-materialism empathy targets received significantly lower empathy than low-materialism empathy targets. Study 2 introduced two control groups and revealed that the empathy difference was due to a reduction in empathy toward high-materialism targets, rather than an increase in empathy toward low-materialism targets. Together with Study 1, this strongly supported H1. Study 3 controlled for social class as a potential confounding variable and ruled out SES as an alternative explanation, further supporting the independent effect of materialism on observer empathy. Study 4 further explored the psychological mechanisms underlying this effect, confirming H2 and H3: the negative impact of target materialism on observer empathy was fully mediated by perceived morality and perceived warmth. Together, these findings provide converging evidence that people selectively downregulate their empathy toward targets perceived as materialistic, and they do so partly because they view such individuals as less moral and less socially warm.

### 9.1. Contributions to Theory

First, our findings significantly extend the theoretical understanding of selective empathy by explicitly integrating materialism as a novel antecedent within the framework of the Motivated Empathy Model (Zaki, 2014). According to Zaki (2014), empathy is not simply an automatic emotional response, but rather involves deliberate cognitive evaluations of the target individual’s characteristics and motivational factors. Previous research within this theoretical tradition has primarily focused on selective empathy toward targets who differ in moral standing (Riva et al., 2016), socioeconomic status (Guo et al., 2012; Lucas & Kteily, 2018), group membership (Brown et al., 2006), or physical attractiveness (Kopiś et al., 2020; Müller et al., 2013). For example, Riva et al. (2016) demonstrated that individuals selectively downregulate empathy when confronted with morally objectionable targets, because such targets evoke reduced perceptions of moral worth. Similarly, Lucas and Kteily (2018) showed that targets of higher socioeconomic status tend to evoke less empathy due to perceived lower need and reduced similarity.

Our findings add to this body of knowledge by demonstrating that observers also selectively downregulate their empathy toward individuals perceived as materialistic. This finding is particularly notable because materialism—unlike overt moral transgressions—does not necessarily imply explicit wrongdoing. Instead, materialism functions as a more subtle social–cognitive cue, indicating that the target individual prioritizes material possessions and external rewards over communal relationships and social connections (Kasser & Ryan, 1993; Richins & Dawson, 1992). Our results show clearly that observers are sensitive to such cues, reducing their empathic concern toward individuals whose value orientations they perceive as misaligned with communal and prosocial ideals.

By identifying materialism as a meaningful antecedent of empathic regulation, this research provides novel insights into the underlying cognitive and motivational processes that drive selective empathy. More specifically, we demonstrate that empathy regulation does not require overt moral violations or explicit out-group membership to occur; subtle differences in value orientations (such as materialism) can be sufficient. Thus, our research contributes to the motivated empathy literature (Cameron et al., 2019; Zaki, 2014) by significantly broadening the scope of recognized social and moral cues that observers utilize when regulating their empathic responses.

Second, our findings deepen and broaden the theoretical understanding of the interpersonal consequences of materialism, extending the literature beyond the traditional focus on intrapersonal psychological outcomes. Prior research has thoroughly documented the negative intrapersonal implications of holding materialistic values, such as reduced life satisfaction, lower subjective well-being, increased depression and anxiety, and impaired social relationships (Dittmar et al., 2014; Kasser, 2016; Ryan & Dziurawiec, 2001). For example, Kasser (2016) argued that materialistic individuals tend to suffer psychologically due to the misalignment between their extrinsic pursuits and intrinsic human needs for social connection and autonomy. Similarly, studies consistently reveal that materialists experience reduced relationship quality, increased interpersonal conflict, and decreased social connectedness (Solberg et al., 2004; Richins, 1994).

However, despite the extensive intrapersonal focus of past research, relatively little is known about how materialistic individuals are perceived and emotionally responded to by others in social interactions. Addressing this gap, our study adopts an observer-centric approach to examine how materialistic individuals are socially evaluated and subsequently empathized with by others. We demonstrate empirically that materialistic individuals, when observed by others, tend to be morally devalued and socially distrusted, leading to significantly reduced empathy toward them. This provides novel evidence that materialism not only harms the individuals who hold these values but also has substantial interpersonal repercussions in terms of social perceptions and empathic responses.

By emphasizing these interpersonal dynamics, our research significantly advances current understandings of the social consequences of materialism (Van Boven et al., 2010; Gentina et al., 2018a). Van Boven et al. (2010), for example, indicated that people generally hold negative stereotypes of materialists, viewing them as self-centered and superficial. Our study substantially extends this insight by explicitly linking these negative stereotypes—specifically perceived morality and warmth—to empathy regulation processes. Thus, we demonstrate that negative interpersonal consequences of materialism not only involve abstract judgments or stereotypes, but also extend to concrete emotional responses, shaping the very empathic fabric of interpersonal relationships. By incorporating an observer perspective and empirically linking materialism to reduced empathic responses from others, our findings considerably enrich and expand the theoretical discourse on materialism, moving beyond intrapersonal psychological consequences and explicitly highlighting its significant interpersonal repercussions.

Third, our research offers a novel theoretical integration by applying the Big Two Model of social perception (Abele & Hauke, 2020; Abele & Wojciszke, 2007) to the domain of materialism and empathy. According to the Big Two Model, interpersonal evaluations predominantly revolve around two fundamental dimensions: morality (e.g., integrity, trustworthiness, and fairness) and warmth (e.g., friendliness, empathy, and interpersonal connectedness). Extensive prior research has shown that these two dimensions strongly influence social judgments, interpersonal attraction, and emotional responses toward others (Brambilla et al., 2021; Fiske et al., 2007; Wojciszke et al., 2009). For instance, Brambilla et al. (2021) argued that perceived morality strongly determines trust, respect, and willingness to engage in prosocial behaviors toward targets, while Fiske et al. (2007) consistently documented that perceived warmth shapes emotional reactions and interpersonal engagement.

However, previous applications of the Big Two Model have largely focused on broad social stereotypes (e.g., competence versus warmth in stereotyping), leadership evaluation, or direct moral transgressions (Abele & Hauke, 2020; Brambilla & Riva, 2017). The current research substantially extends this framework by applying these dimensions to judgments of individuals’ materialistic orientations—an area that had not been explicitly addressed within this theoretical paradigm. Our findings clearly demonstrate that perceived morality and warmth mediate the relationship between target materialism and observer empathy. Specifically, observers judged materialistic targets as lower in morality (e.g., less fair and less trustworthy) and lower in warmth (e.g., less friendly and less interpersonally engaging), which in turn reduced empathic responses toward these individuals.

By empirically confirming that morality and warmth operate as key psychological mediators linking materialism to observer empathy, our findings provide direct support for the relevance and generalizability of the Big Two dimensions in understanding social evaluations toward materialistic individuals. Moreover, this research suggests that these two dimensions—previously applied primarily to broader stereotyping or direct moral contexts—can also meaningfully inform nuanced judgments about personal values and value-driven behaviors, such as materialistic consumption and self-presentation.

Thus, the current study enriches the theoretical scope of the Big Two Model by demonstrating its applicability to a new context: interpersonal reactions to materialistic values and behaviors. This contributes to a more comprehensive understanding of how universal social cognitive dimensions such as morality and warmth fundamentally shape empathic interactions, social evaluations, and ultimately, interpersonal relationships.

Fourth, our study also expands social exchange theory (Blau, 2017; Cropanzano & Mitchell, 2005) by applying it to the relationship between materialism and empathy regulation. Social exchange theory argues that interpersonal relationships and emotional investments are driven by implicit cost–benefit analyses, whereby people prefer to engage emotionally and behaviorally with those perceived as offering higher potential rewards or lower relational costs (Blau, 2017). This perspective has been extensively used to explain social behavior across various domains, such as organizational relationships (Cropanzano & Mitchell, 2005), helping behaviors (Flynn, 2003), and interpersonal trust formation (Colquitt & Rodell, 2011). Yet, despite its widespread applications, social exchange theory has seldom been explicitly applied to explain empathy. Our study demonstrates that empathic responses can indeed reflect an underlying implicit social exchange calculus in some cases. Specifically, observers perceive materialistic individuals as socially less rewarding interaction partners because of their lower perceived morality and warmth. This negative evaluation transforms materialistic targets into “high-cost, low-benefit” partners, resulting in reduced empathic investment from observers. By empirically confirming perceived morality and warmth as crucial evaluative dimensions that determine empathic investments, our findings show clearly that empathy is not merely an automatic emotional response but also involves implicit social exchange considerations. Our study thus expands social exchange theory by suggesting that emotional investments such as empathy are similarly subject to the implicit relational calculus of anticipated social reward and cost. This novel integration enhances theoretical understanding of why certain individuals—specifically those with materialistic values—experience disadvantage in social and emotional interactions.

By applying social exchange theory to the intersection of empathy and materialism, we illuminate a previously unexplored aspect of empathic interaction: observers’ implicit cost–benefit evaluations of targets’ moral and social characteristics. This significantly broadens the explanatory power and applicability of social exchange theory, highlighting its value as a theoretical lens for understanding complex emotional dynamics, such as empathy regulation in interpersonal contexts.

### 9.2. Contributions to Practice

First, our findings offer timely and socially relevant insights for reflecting on materialistic value systems. While most previous research has concentrated on the internal psychological costs of materialism—such as reduced well-being and life satisfaction (Kasser, 2016; Dittmar et al., 2014)—our study highlights an often-overlooked interpersonal consequence: materialistic individuals are likely to receive less empathy from others, even in times of misfortune. This shift in perspective encourages not only individual reflection but also collective re-evaluation of societal values, especially in consumer-driven cultures where material success is frequently celebrated and emulated (Twenge & Kasser, 2013). In this regard, our study provides empirical support for public efforts—especially in educational and cultural spheres—to promote intrinsic goals such as community, personal growth, and emotional connection, rather than material acquisition, as the foundation of well-being and social harmony.

Second, this research provides important implications for personal and organizational impression management in an era dominated by digital self-presentation. On social media platforms, individuals frequently engage in conspicuous consumption to convey success and desirability (Chae, 2018; Jin & Ryu, 2020). However, our findings indicate that such materialistic self-presentation may backfire—eliciting negative social evaluations and reducing observers’ willingness to emotionally invest in the individual. This has practical relevance for influencers, public figures, and everyday users who wish to cultivate genuine trust and connection in online spaces. Instead of prioritizing material symbols, our results suggest that expressing moral concern, emotional authenticity, and interpersonal warmth may be more effective strategies to build positive impressions.

### 9.3. Limitations and Future Directions

Although this study provides initial evidence of the negative impact of materialism on observer empathy and identifies its underlying psychological mechanisms (perceived morality and perceived warmth), there are several limitations that should be addressed in future research. First, we manipulated materialism perception using text-based descriptions based on the three core dimensions of materialism proposed by (Richins & Dawson, 1992): Centrality of possessions, material possessions as a marker of success, and material wealth as a means to happiness. This approach aligns with Zhang and Mick’s (2019) manipulation of materialism perception. While manipulation checks confirmed its effectiveness, it may not fully capture how materialism is perceived in real-life interactions. Future research could explore alternative manipulation methods (e.g., using images, videos, or real-life scenarios) to enhance ecological validity. Second, this study primarily focused on empathy in response to misfortune (negative empathy). However, empathy is not limited to negative contexts—there is also positive empathy, which occurs in joyful or pleasurable situations (Morelli et al., 2015). Future studies should examine whether our findings hold in positive situations—specifically, whether the psychological mechanisms underlying reduced empathy toward materialists apply to positive empathy as well. Third, the sample was drawn solely from a collectivist cultural context (China), which may influence how materialism is morally and socially interpreted. Cross-cultural comparisons would help determine the generalizability of the findings across different cultural frameworks. Fourth, this study relied primarily on affective empathy as measured by self-report. Incorporating complementary assessments—such as cognitive empathy, behavioral indicators, or physiological responses—could offer a more comprehensive understanding of empathic processes. Fifth, individual differences among observers were not measured, yet variables such as observers’ own materialism, moral identity, or social dominance orientation may moderate empathic reactions. Finally, although multiple misfortune scenarios were used, the artificial nature of vignette-based methods may still limit the richness of contextual information. Future studies might employ more interactive or dynamic paradigms, such as VR-based empathy tasks or real-time decision-making simulations. Taken together, addressing these limitations will strengthen future research and deepen our understanding of empathy regulation in response to value-based social judgments.

## 10. Conclusions

This study explored the impact of the materialism of the empathy target on observer empathy and found that higher materialism significantly reduces observer empathy toward materialists by lowering perceived morality and perceived warmth. This finding provides a new perspective on the relationship between materialism and empathy and further validates the application of the “Big Two Model” in social cognition in understanding the interaction between empathy and materialism, and expands our understanding of the selectivity of empathy.

## Figures and Tables

**Table 1:** Descriptive Statistics, ANOVA Results, and Post Hoc Comparisons for Empathy Across Four Materialism Conditions (Study 2).

Group	*N*	Mean	*SD*	ANOVA	Post Hoc (LSD) Comparisons (Mean Difference [*p*])
Low	88	27.08	3.84	*F*(3, 358) = 16.46 *p* < 0.001 η*_p_*^2^ = 0.121	High vs. mod = 2.52 *** (*p* < 0.001)
Moderate	93	26.48	4.52		High vs. none = 4.41 *** (*p* < 0.001)
High	94	23.97	5.54		Low vs. mod = 0.60 (*p* = 0.36)
None	87	28.38	3.42		Low vs. none = 1.30 (*p* = 0.049)

(***: *p* < 0.001).

**Table 2:** Descriptive Statistics and Two-Way ANOVA Results for Observer Empathy (Study 3).

Ma	So	*N*	*M*(e)	*SD*	Source	SS	*df*	MS	*F*	*p*	η^2^*_p_*
Low	Low	97	27.81	4.61	Ma	733.22	1	733.22	19.00	<0.001	0.049
Low	High	96	24.07	6.54	So	718.90	1	718.90	18.63	<0.001	0.048
High	Low	88	24.05	6.91	In	88.15	1	88.15	2.29	0.132	0.006
High	High	94	22.24	6.60					—	—	—

Note: Ma = materialism; So = social class; *M*(e) = mean of empathy; source = ANOVA source; and In = interaction.

**Table 3:** Multivariate and Univariate Analysis of Variance (MANOVA) Results for Study 4.

Variable	Low Materialism *M*(*SD*) *N* = 389	High Materialism *M*(*SD*) *N* = 396	*F*	*p*	η*_p_*^2^
Empathy	29.41 (3.14)	25.42 (6.49)	119.6	<0.001	0.133
Perceived Morality	17.06 (1.99)	11.82 (4.06)	524.23	<0.001	0.401
Perceived Warmth	25.50 (3.01)	17.23 (6.09)	577.98	<0.001	0.425

**Table 4:** Mediation Analysis of the Effects of Target Materialism on Observer Empathy via Perceived Morality and Perceived Warmth.

Path	*B*	*SE*	*t*/BootSE	*p*	95% CI	*β* (Partially Std.)	% of Total Effect
Total effect	−1.99	0.18	−10.94	<0.001	[−2.35, −1.64]		100%
Direct effect	0.36	0.21	1.73	0.085	[−0.05, 0.76]		
Perceived morality (a_1_)	−2.62	0.11	−22.9	<0.001		−0.63	
Perceived morality → empathy (b_1_)	0.43	0.09	5	<0.001		0.33	
Indirect via morality (a_1_b_1_)	−1.13	0.25			[−1.63, −0.65]	−0.21	48.11%
Perceived warmth (a_2_)	−4.13	0.17	−24.04	<0.001		−0.65	
Perceived warmth → empathy (b_2_)	0.3	0.06	5.14	<0.001		0.34	
Indirect via warmth (a_2_b_2_)	−1.22	0.23			[−1.70, −0.77]	−0.22	51.89%
Total indirect effect	−2.35	0.22			[−2.79, −1.95]		117.90%

## Data Availability

The data that support the findings of this study are openly available in Open Science Framework. https://doi.org/10.17605/OSF.IO/MG7JK.

## Appendix A

Vignettes:

**Vignette 1: High-Materialism Group (Used in Study 1 and Study 2)**

Zhang Hua is an employee at a company. Regarding Zhang Hua, he/she is someone who places great importance on purchasing and owning various items. In fact, possessions and wealth are central to his/her daily life. This includes personal transportation, clothing, household items, and more. In Zhang Hua’s view, having various material possessions is a necessary condition for a happy life. He/she also frequently shares photos of newly purchased items on social media. Zhang Hua believes that shopping brings a lot of joy.

Recently, in order to cut costs, Zhang Hua’s company reduced employee benefits. Zhang Hua’s year-end bonus was halved, and his/her monthly salary was reduced by 30%. This reduction in income has had a significant impact on Zhang Hua’s purchasing power.

**Vignette 2: Low-Materialism Group (Used in Study 1 and Study 2)**

Zhang Hua is an employee at a company. Regarding Zhang Hua, he/she is not someone who places great importance on purchasing and owning various items. In fact, possessions and wealth are not central to his/her daily life. This includes Zhang Hua’s transportation, clothing, household items, and so on. In Zhang Hua’s view, having various material possessions is not a necessary condition for a happy life. He/she rarely shares photos of newly purchased items on social media. Zhang Hua believes that shopping does not bring much joy.

Recently, in order to cut costs, Zhang Hua’s company reduced employee benefits. Zhang Hua’s year-end bonus was halved, and his/her monthly salary was reduced by 30%. This reduction in income has had a significant impact on Zhang Hua’s purchasing power.

**Vignette 3: Moderate-Materialism Group (Used in Study 2)**

Zhang Hua is an employee at a company. Regarding Zhang Hua, he/she holds a balanced attitude towards purchasing and owning various items. While possessions and wealth occupy a certain place in his/her life, they do not constitute the entirety of it. This includes Zhang Hua’s transportation, clothing, household items, and so on, which have both practical value and some degree of satisfaction for him/her. In Zhang Hua’s view, having a certain amount of material wealth can bring convenience and comfort to life, but it does not have a decisive impact on inner happiness. He/she does share photos of newly purchased items on social media, but not too frequently. Zhang Hua believes that moderate shopping can bring a pleasant mood, but excessive material pursuit may lead to frustration.

Recently, in order to cut costs, Zhang Hua’s company reduced employee benefits. Zhang Hua’s year-end bonus was halved, and his/her monthly salary was reduced by 30%. This reduction in income has had a significant impact on Zhang Hua’s purchasing power.

**Vignette 4: No Description of Materialism Group (Used in Study 2)**

Zhang Hua is an employee at a company. Recently, in order to cut costs, Zhang Hua’s company reduced employee benefits. Zhang Hua’s year-end bonus was halved, and his/her monthly salary was reduced by 30%. This reduction in income has had a significant impact on Zhang Hua’s purchasing power.

**The Four Vignettes for Study 3:**

**Vignette A: High-Materialism, High-Social-Class Group**

Zhang Hua is an executive at a manufacturing company, earning an annual salary of 1 million yuan. Zhang Hua places great importance on purchasing and owning various material possessions. In fact, possessions and wealth are central to his/her daily life, including transportation, clothing, household items, and more. Zhang Hua believes that owning a variety of material wealth is a necessary condition for a happy life. He/She often shares newly purchased items on social media. Zhang Hua believes that shopping brings a lot of joy. However, recently, in order to cut costs, Zhang Hua’s company reduced the benefits for company executives. Zhang Hua’s year-end bonus was halved, and his/her monthly salary was reduced by 30%. This reduction in income has had a significant impact on Zhang Hua’s purchasing power.

**Vignette B. High-Materialism, Low-Social-Class Group**

Zhang Hua is a worker at a manufacturing company, earning an annual salary of 50,000 yuan. Zhang Hua places great importance on purchasing and owning various material possessions. In fact, possessions and wealth are central to his/her daily life, including transportation, clothing, household items, and more. Zhang Hua believes that owning a variety of material wealth is a necessary condition for a happy life. He/She often shares newly purchased items on social media. Zhang Hua believes that shopping brings a lot of joy. However, recently, in order to cut costs, Zhang Hua’s company reduced the benefits for the workers. Zhang Hua’s year-end bonus was halved, and his/her monthly salary was reduced by 30%. This reduction in income has had a significant impact on Zhang Hua’s purchasing power.

**Vignette C. Low-Materialism, High-Social-Class Group**

Zhang Hua is an executive at a manufacturing company with an annual salary of 1 million yuan. However, Zhang Hua is not someone who places great importance on purchasing and owning various material possessions. In fact, possessions and wealth are not central to his/her daily life. This includes transportation, clothing, household items, and more. Zhang Hua believes that having material wealth is not a necessary condition for a happy life. He/She rarely shares newly purchased items on social media. Zhang Hua believes that shopping does not bring significant happiness. Recently, in order to cut costs, Zhang Hua’s company reduced benefits for executives. Zhang Hua’s year-end bonus was halved, and his/her monthly salary was reduced by 30%. This reduction in income has had a significant impact on Zhang Hua’s purchasing power.

**Vignette D. Low-Materialism, Low-Social-Class Group**

Zhang Hua is a worker at a manufacturing company with an annual salary of 50,000 yuan. However, Zhang Hua is not someone who places great importance on purchasing and owning various material possessions. In fact, possessions and wealth are not central to his/her daily life. This includes transportation, clothing, household items, and more. Zhang Hua believes that having material wealth is not a necessary condition for a happy life. He/She rarely shares newly purchased items on social media. Zhang Hua believes that shopping does not bring significant happiness. Recently, in order to cut costs, Zhang Hua’s company reduced benefits for workers. Zhang Hua’s year-end bonus was halved, and his/her monthly salary was reduced by 30%. This reduction in income has had a significant impact on Zhang Hua’s purchasing power.

**Vignettes for study 4**

**Note: A1–A2 represent the low-materialism and high-materialism descriptions, respectively. B1–B4 are the four different unfortunate events the characters encounter. The materials each participant reads consist of one description from category A and one event from category B.**

A1. Low-materialism: Zhang Hua is a person who values inner experiences. He does not pursue material enjoyment or possession, but focuses more on his spiritual world. He enjoys contemplating the meaning and value of life and cares about the growth of his emotions and thoughts. He rarely spends time and energy on purchasing items, preferring instead to dedicate his time to reading, traveling, and exploring new things. He believes that true happiness comes from inner fulfillment, rather than from the accumulation of material wealth. He does not boast about his purchases or consumption on social media, but maintains a modest and humble attitude. Zhang Hua believes that life is not just about material satisfaction, but also about emotional, spiritual, and social needs. He places more importance on the experiences and development of these aspects than on pursuing material prosperity.

A2. High-materialism: Zhang Hua is a person filled with material desires. He believes that owning various items and wealth is a necessary condition for a happy life. He often shows off the new items he has purchased on social media, using the quantity and brand of items to display his status and success. He views an increase in wealth as a reward for his hard work, so he always strives to work harder to earn more income and bonuses. He often reduces other expenses, such as entertainment and travel, in order to purchase the items he likes. For him, buying and owning things is the most important aspect of his life and a way for him to feel a sense of accomplishment. Even though he is not a very wealthy person, he does his best to buy the things he likes because he believes this will make him happier and more satisfied.

B1. Stock investment failure. One day, Zhang Hua heard that a friend had invested in a stock and made a double return in a short period of time. Inspired by this, Zhang Hua decided to follow his friend’s lead and invest in the same stock. However, the stock soon began to decline. Zhang Hua was horrified to discover that not only had he not made a profit, but he had also lost a significant amount of money. He felt very disheartened, began to doubt his own judgment, and became anxious about the future prospects.

B2. Loss of a grandfather’s only photograph. Zhang Hua has always treasured the only photo left by his grandfather, as it is the only memento he has of him. However, during his recent move, he accidentally lost the photo. He felt deeply upset and saddened because the photo meant so much to him. He wanted to find it, but he knew it was nearly impossible, which made him feel helpless and frustrated.

B3. Diagnosis of an illness. During a recent physical check-up, Zhang Hua was informed that there was an issue with his health that required further examination and treatment. After the tests, he was diagnosed with a long-term illness that required ongoing treatment and regular hospital visits. As a result, Zhang Hua had to adjust his lifestyle, cutting back on work hours and adhering to a strict treatment plan. This had a significant impact on his life, as he had to frequently go to the hospital while also facing the uncertainty and anxiety brought on by the illness. All of this caused him considerable pain and unease.

B4. Stolen phone with the loss of important data. One day, Zhang Hua’s phone was stolen, and he felt very distressed and helpless. His phone contained a large amount of important information, including contacts, work documents, and more. Losing these data would have a significant impact on both his work and personal life. He began to feel that his privacy had been violated, as he had no idea whether his personal information would be leaked.
